# Effects of bisoprolol combined with torasemide on cardiac electrophysiology in patients with acute myocardial infarction and heart failure

**DOI:** 10.3389/fphys.2025.1629758

**Published:** 2025-08-06

**Authors:** Li Jing, Qiangwei Shi, Shihao Zhao

**Affiliations:** Department of Cardiovascular Medicine, The First Affiliated Hospital of Zhengzhou University, Zhengzhou, Henan, China

**Keywords:** acute myocardial infarction, heart failure, bisoprolol, torasemide, cardiac electrophysiology, cardiac function

## Abstract

**Objective:**

This paper aims to assess the impact of bisoprolol combined with torasemide on cardiac electrophysiological parameters in acute myocardial infarction (AMI) patients complicated by heart failure (HF).

**Methods:**

A total of 140 AMI-HF patients were randomized to either the control group (bisoprolol alone for 3 months, n = 70) or an experimental group (bisoprolol for 3 months plus torasemide for 2 weeks, n = 70). The corrected QT (QTc) interval, QT dispersion, B-type natriuretic peptide (BNP) levels, left ventricular ejection fraction (LVEF), serum creatinine, serum potassium, New York Heart Association (NYHA) classification, Borg dyspnea score, lower-limb edema resolution, systolic/diastolic blood pressure (SBP/DBP), heart rate (HR), incidence of ventricular arrhythmia (VA) and atrial flutter/fibrillation (AFL/AF), and cardiovascular and all-cause mortality were evaluated.

**Results:**

Both groups demonstrated statistically significant reductions in QTc interval, QT dispersion, BNP, NYHA class, Borg score, SBP, DBP, HR, along with increased LVEF at 2 weeks and 3 months post-treatment versus baseline, with the experimental group showing moderate improvement at 2 weeks post-treatment in all parameters than the control group (*P* < 0.05). The experimental group had high serum potassium levels and a greater rate of lower-limb edema-resolution at 2 weeks post-treatment than the control group (*P* < 0.05). No significant differences were observed between groups in cardiovascular or all-cause mortality (*P* > 0.05).

**Conclusion:**

Bisoprolol combined with torasemide improves cardiac electrophysiological parameters, cardiac function, symptoms, and hemodynamics in AMI-HF patients as early as 2 weeks into treatment.

## Introduction

Acute myocardial infarction (AMI) remains a leading cause of cardiovascular morbidity and mortality worldwide (Zheng et al., 2025). It is characterized by myocardial necrosis resulting from acute or sustained coronary ischemia and hypoxia, with persistent chest pain as its hallmark symptom (Liu et al., 2024). Globally, an estimated 3 million individuals experience AMI annually, underscoring its significant clinical burden (Tin et al., 2023). Despite advances in therapeutic strategies, patients with AMI continue to face a significant risk for adverse cardiovascular outcomes (Ma et al., 2024). Among these, heart failure (HF) is one of the most common and severe complications following an initial infarction and is strongly associated with worse in-hospital and long-term prognosis (Ma et al., 2024). Clinically, HF is defined by the heart’s inability to maintain adequate cardiac output, resulting in symptoms such as dyspnea, fatigue, and fluid retention (Roghani et al., 2024). It is typically classified into three phenotypes based on left ventricular ejection fraction (LVEF), with reduced ejection fraction (HFrEF) accounting for up to 60% of cases in developed countries (Brennan et al., 2024). The development of HF post-AMI is primarily driven by extensive cardiomyocyte loss and subsequent scar formation, triggering chronic neurohormonal activation. This includes upregulation of the renin-angiotensin-aldosterone system (RAAS) and the sympathetic nervous system, which ultimately contribute to progressive ventricular remodeling (Takeuchi et al., 2024). The risk of HF and mortality is further elevated in post-AMI patients, especially those with persistent congestion or reduced LVEF (Butler et al., 2024).

Current pharmacological management for AMI involves a comprehensive regimen including antithrombotic agents, β-blockers, lipid-lowering drugs, nitrates, calcium channel blockers, and angiotensin-converting enzyme inhibitors (Dong et al., 2025). Among these, β-blockers play a pivotal role, particularly in patients with HFrEF (Roghani et al., 2024). Substantial clinical evidence supports their efficacy in improving symptoms, reducing rehospitalization rates, and decreasing mortality in HF patients (Roghani et al., 2024). Long-term β-blocker therapy following MI has been associated with an approximate 20% reduction in all-cause mortality (Yndigegn et al., 2024). Large-scale randomized controlled trials and meta-analyses have confirmed the benefits of evidence-based β-blockers—specifically bisoprolol, carvedilol, and metoprolol succinate—in terms of improving symptoms, reversing left ventricular remodeling, reducing hospitalizations and mortality, and enhancing overall survival and quality of life in patients with HFrEF (Hassen et al., 2025).

Diuretics have long been a cornerstone of HF management (Wu et al., 2024). Loop diuretics, prescribed in over 90% of HF cases, are indispensable for alleviating volume overload by reducing both central and peripheral edema (Palazzuoli et al., 2024). They play a dual role in achieving decongestion during acute decompensated HF and maintaining euvolemia in chronic HF (Chopra et al., 2023). Although furosemide remains the most widely used loop diuretic, emerging evidence suggests that torasemide may offer superior clinical benefits, including lower rates of rehospitalization and morbidity (Li et al., 2025). Torasemide has favorable pharmacokinetic and pharmacodynamic properties, such as high oral bioavailability, hepatic metabolism, food-independent absorption, a prolonged half-life, and a rapid onset of action (Chopra et al., 2023). Furthermore, it may exert antifibrotic effects on the myocardium, mitigate ventricular hypertrophy and dilation, and exhibit mineralocorticoid receptor antagonistic activity. These attributes have been associated with improved New York Heart Association (NYHA) functional class and a lower incidence of hypokalemia (Palazzuoli et al., 2024).

Although bisoprolol and torasemide are both widely utilized in clinical settings, evidence regarding their combined effects on cardiac electrophysiology in AMI patients complicated by HF remains scarce. Therefore, this study aims to assess the efficacy of bisoprolol in conjunction with short-term torasemide therapy in enhancing electrophysiological parameters, improving cardiac function and clinical symptoms, and ultimately optimizing outcomes in this high-risk population. The findings may offer new insights into the clinical benefits of this combination therapy and support its potential application in routine practice.

## Materials and methods

### Ethics statement

This study was approved by the Ethics Committee of The First Affiliated Hospital of Zhengzhou University. Written informed consent was obtained from all participants prior to enrollment.

### Subjects

A total of 140 patients diagnosed with AMI complicated by HF and admitted to our hospital between May 2022 and December 2024 were included in this study.

Inclusion criteria were as follows: (1) Hospitalized patients meeting the diagnostic criteria for AMI complicated by HF (McDonagh et al., 2021); (2) Aged 18–80 years, with NYHA cardiac function class II-IV; (3) Provided written informed consent and voluntarily participated in the study and follow-up; (4) Baseline electrocardiogram suitable for measurement of corrected QT (QTc) interval and QT dispersion, without severe conduction abnormalities (e.g., third-degree atrioventricular block), with systolic blood pressure (SBP) ≥ 90 mmHg and heart rate (HR) ≥ 50 bpm; (5) Approval by the hospital’s ethics committee.

Exclusion criteria included: (1) HF secondary to non-ischemic cardiomyopathy, significant valvular heart disease, or congenital heart disease; (2) Severe hepatic or renal dysfunction; (3) Known hypersensitivity or intolerance to bisoprolol, torasemide, or related drugs; (4) Pregnancy or lactation; (5) Active malignancy, ongoing severe infection, autoimmune disease, or other terminal illnesses; (6) Cardiac resynchronization therapy (CRT) or implantable cardioverter-defibrillator (ICD) implantation within 3 months prior to enrollment; (7) Contraindications to β-blockers (e.g., asthma, severe bradycardia, uncontrolled hypotension); (8) Poor treatment compliance (e.g., irregular medication use or follow-up); or concurrent participation in other clinical trials.

### Randomization and blinding procedures

Following baseline screening, patients were randomly assigned in a 1:1 ratio to either Group A (control group) or Group B (experimental group) using a computer-generated randomization sequence. Allocation was concealed, and investigators remained blinded to group assignments until randomization was complete. Outcome assessments were conducted by trained research assistants who were blinded to treatment allocation.

### Treatment methods

All enrolled patients received standard medical management, including bed rest, supplemental oxygen, vasodilators, and fluid volume management, along with guideline-directed medical therapy for heart failure. This included angiotensin receptor neprilysin inhibitors, mineralocorticoid receptor antagonists, and sodium-glucose cotransporter-2 inhibitors.

(1) Group A (control group): Patients received the following conventional medications:

Sacubitril/Valsartan Sodium Tablets (Novartis Farma S.p.A.; 100 mg; approval number: HJ20170362): Initiated at 50 mg twice daily. After 7 days, tolerance was assessed. If well tolerated and effective, the dose was titrated up to 100 mg twice daily.

Spironolactone Tablets (Guangdong Huanan Pharmaceutical Group Co., Ltd.; NMPA Approval No. H44020686): 10 mg twice daily.

Dapagliflozin Tablets (AstraZeneca Pharmaceutical Co., Ltd.; NMPA Approval No. J20170040): Started at 5 mg once daily, with dose escalation to 10 mg once daily after 7 days if tolerated.

Bisoprolol Fumarate Tablets (Beijing Hisun Pharmaceutical Co., Ltd.; NMPA Approval No. H20023132): Initiated at 1.25 mg once daily, with gradual uptitration based on tolerance and clinical status, up to a maximum of 10 mg once daily.

Patients first received bisoprolol and dapagliflozin, followed by the addition of sacubitril/valsartan and spironolactone within 7 days. The total duration of treatment was 3 months.

(2) Group B (experimental group): In addition to the medications received by the control group, patients in the experimental group also received Torasemide. Torasemide (Biocause Heilen Pharmaceutical Co., Ltd., Hubei, China; NMPA Approval No. H20040074; 5 mg/tablet): Administered orally at an initial dose of 10 mg once daily in the morning. The dose was adjusted between 5 and 20 mg/day according to the degree of edema and renal function. Torasemide therapy was maintained for 2 weeks.

### Observation indicators

(1) Cardiac electrophysiological indicators: A standard 12-lead electrocardiogram (ECG; Philips PageWriter TC70) was used to measure the corrected QT (QTc) interval—calculated using the Bazett formula (QTc = QT/√RR) with a filter range of 0.05–150 Hz—and QT dispersion, defined as the difference between the maximum and minimum QT intervals across leads in the same ECG. All measurements were averaged over three consecutive cardiac cycles and were assessed at baseline, and at 2 weeks and 3 months post-treatment.
(2) Cardiac function indicators and electrolyte status: Fasting venous blood samples (2 mL) were collected at baseline and at 2 weeks and 3 months post-treatment to determine serum B-type natriuretic peptide (BNP) levels via chemiluminescence immunoassay (RayBiotech, Guangzhou, China; reference range: 0–100 pg/mL). Left ventricular ejection fraction (LVEF) was calculated using Simpson’s biplane method by echocardiography (Philips Affiniti 70). Serum potassium and creatinine levels were analyzed using an automated analyzer (Roche Diagnostics, Burgess Hill, UK) after centrifugation.
(3) NYHA cardiac function classification: Cardiac function was classified at baseline and at 2 weeks and 3 months post-treatment according to the NYHA classification criteria (Bennett et al., 2002): class I (no limitation of physical activity), class II (slight limitation), class III (marked limitation), and class IV (inability to perform any physical activity). For analysis, these were numerically coded as 1, 2, 3, and 4, respectively.
(4) Symptoms and signs: Dyspnea was assessed at baseline and at 2 weeks and 3 months post-treatment using the Borg scale (Borg, 1982), with scores ranging from 0 (no dyspnea) to 10 (maximum dyspnea). The resolution rate of lower limb edema—defined as complete resolution or ≥50% improvement of pitting edema—was also recorded at each time point.
(5) Hemodynamic indicators: SBP, diastolic blood pressure (DBP), and HR were measured at baseline and at 2 weeks and 3 months post-treatment using a calibrated electronic sphygmomanometer (Philips: SureSigns VS3).
(6) Complications: The incidence of ventricular arrhythmia (VA) (Definition of VA, 2022) and atrial flutter/fibrillation (AFL/AF) was recorded at baseline and at 2 weeks and 3 months post-treatment.
(7) Prognosis and endpoint events: Cardiovascular mortality and all-cause mortality were recorded during the treatment period in both groups.

### Statistical analysis

All statistical analyses were performed using SPSS 27.0 software. Categorical variables were expressed as n (%) and compared using the chi-square test or Fisher’s exact test, as appropriate. The Kolmogorov-Smirnov test was applied to assess normality of distribution for continuous variables. Continuous variables with a normal distribution were expressed as mean ± standard deviation (SD) and compared using the independent samples t-test (between groups) or paired t-test (within groups). Non-normally distributed continuous variables were presented as median [interquartile range, IQR] and compared using the Mann-Whitney U test (between groups) or Wilcoxon signed-rank test (within groups). Survival outcomes (mortality) were analyzed using Kaplan-Meier curves and compared with the log-rank test. A two-tailed *P*-value <0.05 was considered statistically significant.

## Results

### Baseline characteristics

The control group and the experimental group each consisted of 70 patients. As shown in Table 1, there were no statistically significant differences between the two groups in baseline characteristics, including age, body mass index, disease duration, gender, history of hypertension, diabetes, or hyperlipidemia, site of myocardial infarction, and Killip classification (all *P* > 0.05), indicating comparability.

**TABLE 1:** Comparison of baseline characteristics between the two groups.

Variable	Experimental group (n = 70)	Control group (n = 70)	*Z/χ* ^2^	*P-value*
Age (years)	62.00 (55.00, 69.00)	61.00 (53.75, 70.00)	−0.273	0.785
Body mass index (kg/m^2^)	26.01 (23.71, 28.29)	25.39 (23.13, 27.61)	−1.436	0.151
Male [n (%)]	42 (60.00)	45 (64.29)	0.273	0.601
Hypertension [n (%)]	49 (70.00)	46 (65.71)	0.295	0.587
Diabetes [n (%)]	20 (28.57)	23 (32.86)	0.302	0.583
Hyperlipidemia [n (%)]	41 (58.57)	39 (55.71)	0.117	0.733
Site of myocardial infarction [n (%)]			0.749	0.688
Anterior wall	25 (35.71)	30 (42.86)	-	-
Inferior wall	26 (37.14)	23 (32.86)	-	-
Others	19 (27.14)	17 (24.29)	-	-
Killip classification [n (%)]			0.922	0.820
Class I	5 (7.14)	8 (11.43)	-	-
Class II	36 (51.43)	33 (47.14)	-	-
Class III	22 (31.43)	23 (32.86)	-	-
Class IV	7 (10.00)	6 (8.57)	-	-

### Cardiac electrophysiological parameters

There were no significant differences in QTc interval and QT dispersion between the two groups before treatment (*P* > 0.05). At both 2 weeks and 3 months post-treatment, QTc interval and QT dispersion decreased in both groups compared to baseline, and the experimental group showed lower QTc interval and QT dispersion than the control group at 2 weeks post-treatment (*P* < 0.05) (Table 2).

**TABLE 2:** Comparison of cardiac electrophysiological parameters between the two groups (ms).

Variable	Group	Before treatment	After 2 weeks post-treatment	After 3 months post-treatment
QTc interval	Experimental group (n = 70)	481.00 (455.50, 507.50)	446.00 (424.75, 468.50)^a^	430.00 (412.75, 450.50)^a^
	Control group (n = 70)	480.00 (454.00, 508.25)	462.00 (436.75, 488.00)^a^	438.50 (419.75, 460.00)^a^
	*Z*	−0.119	−2.939	−1.811
	*P-value*	0.905	0.003	0.070
QT dispersion	Experimental group (n = 70)	91.00 (73.75, 108.50)	60.50 (49.00, 74.25)^a^	45.50 (40.00, 53.00)^a^
	Control group (n = 70)	91.00 (72.50, 111.00)	76.00 (59.50, 94.00)^a^	49.50 (44.00, 56.50)^a^
	*Z*	−0.035	−4.183	−1.906
	*P-value*	0.972	<0.001	0.057

Note: ^a^ *P* < 0.05 vs. same group before treatment. QTc, corrected QT.

### Cardiac function parameters and electrolyte conditions

There were no significant differences in pre-treatment BNP and LVEF between the two groups before treatment (*P* > 0.05). At 2 weeks and 3 months post-treatment, BNP levels in both groups were lower and LVEF values were higher compared with baseline. Furthermore, the experimental group demonstrated moderate improvements in BNP and LVEF at 2 weeks post-treatment compared with the control group (*P* < 0.05). No significant difference was observed in the potassium and creatinine levels between the experimental group and the control group before treatment (*P* > 0.05). After 2 weeks of treatment, the potassium level in the experimental group was lower than that in the control group (*P* < 0.05), while the difference in creatinine levels between the two groups was not significant (*P* > 0.05) (Table 3).

**TABLE 3:** Comparison of cardiac function indicators between the two groups.

Variable	Group	Before treatment	After 2 weeks post-treatment	After 3 months post-treatment
BNP (pg/mL)	Experimental group (n = 70)	868.40 ± 141.69	328.27 ± 80.68^a^	289.01 ± 89.97^a^
	Control group (n = 70)	859.43 ± 149.26	493.00 ± 91.58^a^	313.06 ± 97.68^a^
	*t*	0.365	−11.292	−1.515
	*P-value*	0.716	<0.001	0.132
LVEF (%)	Experimental group (n = 70)	35.00 (30.75, 40.00)	42.00 (37.75, 46.00)^a^	46.00 (42.75, 51.00)^a^
	Control group (n = 70)	35.00 (31.00, 40.00)	39.00 (35.00, 44.00)^a^	44.50 (39.75, 50.00)^a^
	*Z*	−0.559	−2.536	−1.805
	*P-value*	0.576	0.011	0.071
Creatinine	Experimental group (n = 70)	83.46 ± 5.29	80.49 ± 4.47^a^	-
	Control group (n = 70)	83.50 ± 5.25	81.36 ± 4.81^a^	-
	*t*	−0.045	−1.109	-
	*P-value*	0.964	0.270	
Serum potassium	Experimental group (n = 70)	3.79 ± 0.28	3.41 ± 0.50^a^	-
	Control group (n = 70)	3.81 ± 0.37	3.23 ± 0.30^a^	-
	*t*	−0.361	2.583	-
	*P-value*	0.719	0.011	-

Note: ^a^ *P* < 0.05 vs. same group before treatment. BNP, B-type natriuretic peptide; LVEF, left ventricular ejection fraction.

### Cardiac function classification

No notable difference was noted in NYHA cardiac function classification between the two groups prior to treatment (*P* > 0.05). At 2 weeks and 3 months post-treatment, NYHA classification in both groups was improved (i.e., classification score decreased) compared to baseline, with the experimental group showing a lower classification than the control group after 2 weeks of treatment (*P* < 0.05) (Table 4).

**TABLE 4:** Comparison of NYHA functional classification between the two groups(scores).

Group	Before treatment	After 2 weeks post-treatment	After 3 months post-treatment
Control group (n = 70)	3.00 (3.00, 3.00)	2.00 (2.00, 3.00)^a^	2.00 (2.00, 2.00)^a^
Experimental group (n = 70)	3.00 (3.00, 3.00)	3.00 (2.00, 3.00)^a^	2.00 (2.00, 2.00)^a^
*Z*	−0.493	−3.123	−1.213
*P-value*	0.622	0.002	0.225

Note: ^a^ *P* < 0.05 vs. same group before treatment. NYHA, New York Heart Association.

### Symptoms and signs

No significant difference was witnessed in Borg dyspnea scores between the two groups prior to treatment (*P* > 0.05). At 2 weeks and 3 months post-treatment, Borg scores in both groups were lower than before treatment, and the experimental group had lower Borg scores than the control group after 2 weeks of treatment (*P* < 0.05). The resolution rate of lower limb edema in the experimental group was higher than that in the control group after 2 weeks of treatment (*P* < 0.05) (Table 5).

**TABLE 5:** Comparison of symptoms and signs between the two groups.

Variable	Group	Before treatment	After 2 weeks post-treatment	After 3 months post-treatment
Borg dyspnea score (points)	Experimental group (n = 70)	6.00 (5.00, 7.00)	3.00 (3.00, 4.00)^a^	3.00 (2.00, 3.00)^a^
	Control group (n = 70)	6.00 (5.00, 7.00)	4.00 (3.00, 5.00)^a^	3.00 (2.00, 4.00)^a^
	*Z*	−0.334	−4.163	−1.08
	*P-value*	0.738	<0.001	0.28
Resolution rate of lower limb edema [n (%)]	Experimental group (n = 70)	-	53 (75.71%)	62 (88.57%)
	Control group (n = 70)	-	29 (41.43%)	54 (77.14)
	*χ* ^2^	-	16.955	3.218
	*P-value*	-	<0.001	0.073

Note: ^a^ *P* < 0.05 vs. same group before treatment.

### Hemodynamic parameters

There were no differences in SBP, DBP, and HR between the two groups prior to treatment (*P* > 0.05). After 2 weeks and 3 months of treatment, both groups displayed lower SBP, DBP, and HR compared to baseline. Additionally, SBP and DBP in the experimental group were lower than those in the control group after 2-week treatment (*P* < 0.05) (Table 6).

**TABLE 6:** Comparison of hemodynamic parameters between the two groups.

Variable	Group	Before treatment	After 2 weeks post-treatment	After 3 months post-treatment
SBP (mmHg)	Experimental group (n = 70)	135.00 (128.00, 142.00)	119.00 (112.75, 125.00)^a^	117.00 (111.75, 122.25)^a^
	Control group (n = 70)	135.00 (127.75, 144.00)	125.50 (118.75, 133.00)^a^	120.00 (112.00, 126.00)^a^
	*Z*	−0.628	−4.141	−1.587
	*P-value*	0.530	<0.001	0.112
DBP (mmHg)	Experimental group (n = 70)	85.00 (79.75, 91.00)	77.00 (72.75, 81.00)^a^	74.00 (70.00, 78.00)^a^
	Control group (n = 70)	84.00 (77.75, 90.00)	81.00 (76.00, 86.00)^a^	76.00 (71.00, 80.00)^a^
	*Z*	−1.072	−3.537	−1.918
	*P-value*	0.284	<0.001	0.055
HR (bpm)	Experimental group (n = 70)	86.04 ± 8.71	76.06 ± 7.17^a^	73.07 ± 7.19^a^
	Control group (n = 70)	87.00 ± 7.22	80.00 ± 7.22^a^	74.96 ± 6.30^a^
	*t*	−0.708	−3.242	−1.651
	*P-value*	0.480	0.001	0.101

Note: ^a^ *P* < 0.05 vs. same group before treatment. SBP, systolic blood pressure; DBP, diastolic blood pressure; HR, heart rate.

### Complications

There showed no significant differences in the incidence rates of VA and AFL/AF between the two groups at baseline, or at 2 weeks/3 months post-treatment (*P* > 0.05). However, in the experimental group, the incidence rates of VA and AFL/AF following 3-month treatment were lower compared to baseline (*P* < 0.05) (Table 7).

**TABLE 7:** Comparison of comorbidities between the two groups [n (%)].

Comorbidity	Group	Before treatment	After 2 weeks post-treatment	After 3 months post-treatment
VA	Experimental group (n = 70)	14 (20.00)	8 (11.43)	5 (7.14)^a^
	Control group (n = 70)	15 (21.43)	10 (14.29)	7 (10.00)
	*χ* ^2^	0.043	0.255	0.365
	*P-value*	0.835	0.614	0.546
AFL/AF	Experimental group (n = 70)	13 (18.57)	9 (12.86)	3 (4.29)^a^
	Control group (n = 70)	11 (15.71)	8 (11.43)	5 (7.14)
	*χ* ^2^	0.201	0.068	0.530
	*P-value*	0.654	0.796	0.718

Note: ^a^ *P* < 0.05 vs. same group before treatment. VA, ventricular arrhythmia; AFL/AF, atrial flutter/fibrillation.

### Prognosis and endpoint events

**FIGURE 1:**
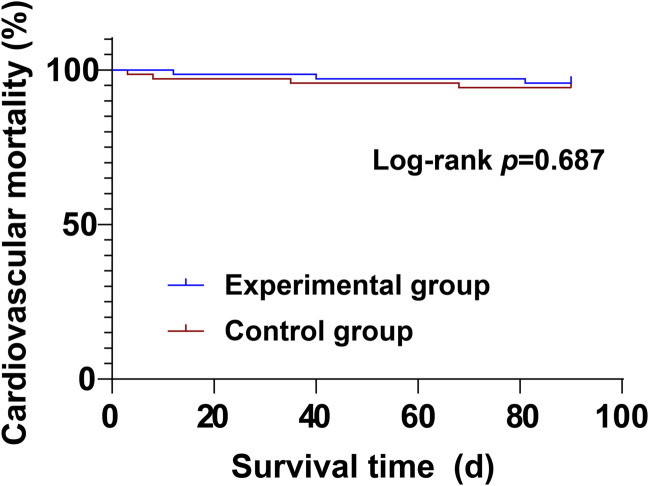
Kaplan-Meier curve of cardiovascular mortality.

**FIGURE 2:**
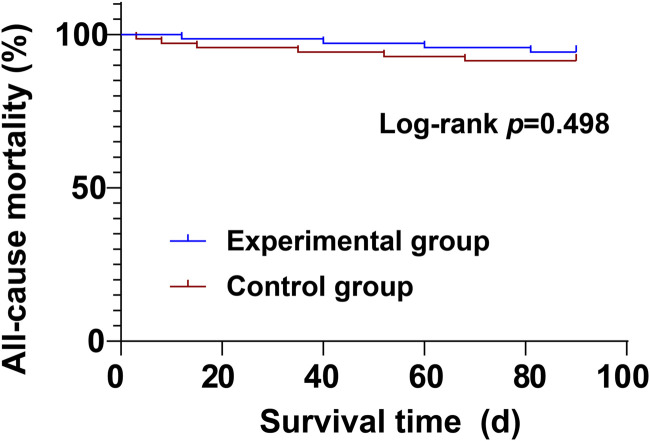
Kaplan-Meier curve of all-cause mortality.

During the treatment period, there were no significant differences between the experimental group and the control group in cardiovascular mortality (4.29% vs. 5.71%) and all-cause mortality (5.71% vs. 8.57%) (*P* > 0.05) (Table 8; Figures 1, 2).

**TABLE 8:** Comparison of prognosis and endpoint events between the two groups [n (%)].

Group	Cardiovascular mortality	All-cause mortality
Experimental group (n = 70)	3 (4.29)	4 (5.71)
Control group (n = 70)	4 (5.71)	6 (8.57)
*χ* ^2^	0.162	0.459
Log-rank *p*	0.687	0.498

## Discussion

We investigated the effects of bisoprolol combined with torasemide on cardiac electrophysiology in patients with AMI complicated by HF. Our findings suggest that this combination therapy can improve cardiac electrophysiological parameters, cardiac function, clinical symptoms, and hemodynamic status after 2 weeks of treatment.

Both groups showed reductions in QTc interval and QT dispersion, with more pronounced improvements observed in the experimental group. Regarding the influence of medications on QTc interval, QT dispersion, and arrhythmia incidence, the review by Koev et al. (2023) on preventing sudden cardiac death provides valuable context. β-blockers are well-established in attenuating arrhythmogenic risk in HF and ischemic heart disease, especially in those with impaired left ventricular function (Cotton et al., 2022). In our study, bisoprolol likely contributed to improved QT parameters through its known antiarrhythmic mechanisms (Nakamura et al., 2016; Li et al., 2011). Torasemide’s antifibrotic properties—including inhibition of type I collagen synthesis via downregulation of procollagen type I carboxy-terminal proteinase—may further homogenize myocardial conduction (Chopra et al., 2023). Myocardial fibrosis disrupts electrical conduction pathways and increases the risk of arrhythmias (Czubryt and Hale, 2021). By attenuating fibrosis, torasemide may improve myocardial structure and conduction, potentially lowering the risk of arrhythmias, which may be reflected in improved QT parameters. However, the precise relationship between these agents and arrhythmia incidence warrants further study. Overall, the observed reductions in QTc interval and QT dispersion may signal enhanced myocardial electrical stability and a reduced arrhythmic risk to some extent.

Furthermore, BNP levels decreased and LVEF improved in both groups, with the experimental group showing more significant improvements at 2 weeks post-treatment. The reduction in BNP indicates decreased cardiac load and improved hemodynamic status, while the increase in LVEF suggests enhanced myocardial contractility. These findings align with the improvements seen in QT parameters, collectively highlighting the benefit of combining bisoprolol and torasemide. Bisoprolol mitigates neurohormonal activation and preserves myocardial integrity (Nakamura et al., 2016), whereas torasemide enhances these effects through potent diuresis and possible direct antifibrotic activity (Chopra et al., 2023). Prior trials have noted torasemide’s superiority over furosemide in augmenting ejection fraction and shortening hospital stays (Mentz et al., 2023). Meta-analyses further support long-term β-blocker use for reducing all-cause and cardiovascular mortality, as well as major adverse cardiac events in post-MI cohorts (Liang et al., 2022). Additionally, large registries have linked β-blocker therapy with improved survival in HFpEF patients following decompensation (Ibrahim et al., 2024). Notably, after 2 weeks of treatment, the experimental group had lower serum potassium levels than the control group, likely due to torasemide’s diuretic effect (K et al., 2025). The absence of a significant difference in creatinine levels suggests that torasemide’s impact on renal function may be relatively modest.

Improvements in NYHA class and Borg dyspnea scores were also more pronounced in the experimental group, alongside a higher rate of edema resolution after 2 weeks. These findings indicate that the combination therapy can effectively alleviate clinical symptoms. Torasemide blocks Na^+^/K^+^/2Cl^-^ cotransporters in the thick ascending limb of Henle—responsible for 20%–30% of filtered sodium reabsorption—thereby promoting natriuresis and diuresis (Palazzuoli et al., 2024). It also exerts reversible inhibition of NKCC2 and acts on distal nephron sites, enhancing fluid removal (Wu et al., 2024).

Both groups experienced significant reductions in SBP, DBP, and HR, with the experimental group exhibiting lower SBP and DBP than the control group. β-blockers reduce HR, myocardial oxygen demand, and adverse sympathetic activity by blocking β-adrenergic receptors (Roghani et al., 2024). Bisoprolol’s efficacy in improving long-term cardiovascular outcomes and blood pressure control has been documented in hypertensive populations (Kim Tran et al., 2023). Although one trial found no superiority of torasemide over furosemide regarding mortality or quality of life post-HF hospitalization (Verbrugge and Menon, 2022), bisoprolol combined with torasemide may producesynergistic antihypertensive effects.

While intergroup differences in VA and AFL/AF incidence were not statistically significant, the experimental group experienced a lower incidence of VA and AFL/AF after 3 months of treatment than at baseline. Torasemide has a relatively long half-life (Mentz et al., 2023), with a faster onset, longer duration of action, and lower risk of abrupt diuresis compared to furosemide (Balsam et al., 2019). These findings may be attributed to torasemide’s ability to maintain effective plasma concentrations over an extended period, thereby sustaining its diuretic effects and reducing cardiac preload and afterload. Even after discontinuation, residual drug levels may continue to exert therapeutic effects. Additionally, the limited sample size in this study may have influenced the observed incidence rate. Long-term β-blocker use after ST elevation myocardial infarction and percutaneous coronary intervention is associated with reduced all-cause mortality (Maqsood et al., 2021). Systematic reviews support β-blockers’ role in decreasing recurrent infarction and long-term mortality, although their impact on short-term mortality remains inconclusive (Safi et al., 2019), especially in patients with preserved LVEF (Sabina et al., 2024). The trend toward reduced arrhythmia incidence in the experimental group may suggest potential long-term survival benefits, but this warrants validation through longer follow-up. Notably, no significant differences in cardiovascular or all-cause mortality were observed between groups during the treatment period. Early initiation of oral carvedilol post-percutaneous coronary intervention has been shown to reduce long-term mortality (Dai et al., 2024), and β-blockers, including bisoprolol, consistently demonstrate survival benefits in HF by attenuating catecholamine effects and preventing arrhythmias (Roghani et al., 2024). Although the TRANSFORM-HF trial found no mortality difference between furosemide and torasemide (Verbrugge and Menon, 2022), meta-analyses have favored torasemide for reducing HF hospitalization rates without a clear effect on mortality (Singh et al., 2023). Given the complex interplay of drug mechanisms and clinical effects, mortality outcomes require further validation in long-term studies.

From a mechanistic perspective, torasemide, as a novel loop diuretic, may offer cardiovascular protection beyond its diuretic action due to its multifaceted properties. Evidence suggests that torasemide can reverse myocardial fibrosis, inhibit type I collagen synthesis, and improve cardiac remodeling in chronic HF patients (Acuna, 2010). These effects are particularly important during post-MI remodeling, as they may reduce the arrhythmogenic substrate by enhancing myocardial structural uniformity. Additionally, torasemide exhibits unique aldosterone antagonistic activity, inhibiting aldosterone binding and downstream signaling (Acuna, 2010; Potter et al., 2019). Aldosterone promotes myocardial fibrosis, inflammation, and oxidative stress by activating mineralocorticoid receptors in cardiomyocytes, thereby increasing arrhythmia risk (Tsai et al., 2024; Khan et al., 2024). When combined with β-blockers, torasemide may synergistically counteract excessive neurohormonal activation, further stabilizing myocardial electrophysiology.

It is important to note that while both furosemide and torasemide are loop diuretics used in HF management (Li et al., 2025), they differ in pharmacokinetic and pharmacodynamic profiles. Torasemide has higher bioavailability and a longer half-life than furosemid (Mentz et al., 2023), resulting in faster onset, sustained action, and a lower risk of rapid diuresis (Balsam et al., 2019). Moreover, torasemide’s anti-aldosterone, vasodilatory, and antifibrotic properties may confer additional cardiovascular benefits beyond fluid removal. In contrast, furosemide primarily acts as a diuretic and natriuretic agent without direct protective effects on myocardial remodeling (Mentz et al., 2023; Balsam et al., 2019). Recent TRANSFORM-HF trials have reported no significant differences in all-cause mortality or HF rehospitalization rates between torasemide and furosemide among patients discharged after HF hospitalization (Kittipibul et al., 2024). Regardless of LVEF or baseline status, patient-reported outcomes appear similar between the two agents (Kapelios et al., 2024; Greene et al., 2023). Future multi-center, large-scale studies are needed to further clarify the comparative benefits of torasemide and furosemide.

In conclusion, bisoprolol combined with torasemide improved cardiac electrophysiological parameters, cardiac function, symptoms, and hemodynamics in patients with AMI complicated by HF after 2 weeks of treatment. This combination regimen may provide clinicians with an additional therapeutic option to optimize management and improve patient outcomes. However, this study has several limitations. First, the short follow-up period precludes assessment of the long-term effects of bisoprolol-torasemide therapy on cardiac electrophysiology, function, and prognosis in AMI-HF patients; extended observation is needed to evaluate its sustained efficacy and safety. Second, the small sample size may introduce bias and limit the generalizability of the findings, particularly for mortality and arrhythmia endpoints. Additionally, metabolic parameters such as blood glucose and lipid profiles were not monitored, potentially overlooking the drugs’ metabolic impact. Future research should extend follow-up duration, expand the sample size, and adopt multi-center, randomized controlled trial designs to enhance external validity. Furthermore, comprehensive monitoring of metabolic markers will help elucidate the full spectrum of effects. Finally, in-depth mechanistic studies exploring how bisoprolol combined with torasemide improves cardiac electrophysiology and function could identify novel therapeutic targets and provide theoretical support for developing more effective treatment strategies.

## Data Availability

The raw data supporting the conclusions of this article will be made available by the authors, without undue reservation.
